# Cutaneous anaplastic large cell lymphoma in HIV exceptional case in Algeria

**DOI:** 10.1186/1471-2334-14-S2-P51

**Published:** 2014-05-23

**Authors:** M Rais, A Ouyahia, W Guenifi, A Gasmi, S Mechakra, A Lacheheb

**Affiliations:** 1 Infectious diseases Hospital, Faculty of medicine of Setif, Setif, Algeria

## Introduction

Anaplastic large cell lymphoma is a rare and account for 10 % of cases of cutaneous lymphoma T is characterized by the presence of anaplastic tumor cells, pleomorphic or immunoblastic for the majority of them (over 75 %) antigen CD30 . It is in the form of single or multiple nodules or as patches in adult patients.

## Patient

We report a patient of 43 years old followed for HIV + hypothyroidism who presented for 8 months multiple purplish nodules scattered all the body but especially in the left lower limb or there is a large ulcerated a budding and painful nodule the upper part of the inner side of the left thigh associated with lymphedema of the lower limb all concerned Clinically, it measured 3-5 cm in diameter, were clear limits, an erythematous ulcerated area with serosanglant flow and was fixed with respect to deeper level. We found nodular lesions on the abdominal and thoracic wall. He had no lymphadenopathy or hepato splenomegaly. We discussed the diagnosis of cutaneous leishmaniasis, epidermoid carcinoma, cutaneous tuberculosis and Kaposi sarcoma. Histological examination of a fragment of skin resection was in favor of anaplastic large T-cell lymphoma / No primitive according to OMS classification 2008 (CD30 + , CD20 - , CD5 + / -, ALK- , CD15 - and granzyme B + / - ). Lymph node ultrasound, thoracoabdominal computed tomography showed thoraco-abdominal polyadenopathies with two right pulmonary micronodules. The treatment consisted in CHOP chemotherapy protocol.

After four cycles of chemotherapy lesions disappeared completely with disappearance of lymphedema with a very good condition and weight regain. Thoracoabdominal CT scan was done showing the complete disappearance of thoraco- abdominal lymphadenopathy.

## Conclusion

Front dragging and recurrent chronic ulcers, the diagnosis of cutaneous lymphoma should be considered in the differential diagnosis and skin biopsy is needed.

